# Mechanisims of asthma and allergic disease – 1068. Why we should not use steroids against infections in infants with atopic dermatitis

**DOI:** 10.1186/1939-4551-6-S1-P65

**Published:** 2013-04-23

**Authors:** Akiyoshi Yamanaka

**Affiliations:** 1 Department of Oriental Medicine, Yurigaoka Clinic, Nabari City Mie Pref., Japan

## Background

Steroids used for allergies have a side effect to exacerbate the infection. However, the short-term steroid use is clinically effective against Atopic dermatitis (AD) infections. One reason is that scratching the eczema increases the risk of infection further. Another reason is that S. aureus exacerbates AD is by secreting toxins called superantigens, which stimulate activation of T cells and macrophages. Clinically, patients with AD, regardless of whether there is a skin infection, respond better to combined treatment with topical corticosteroids and antistaphylococcal antibiotics than to topical steroid therapy alone. In younger infants, their immune system development is more strongly affected by immunosuppressive agents. The present study was designed to investigate the long term effect of steroid use and recovery in siblings, where one sibling had been treated with steroid medication whereas the other sibling hadn't.

## Methods

From 1997 to 2009, 26 pairs of siblings with AD were treated at a clinic, where AD is managed without the use of steroids, and were divided into “users” and “nonusers” groups based on previous steroid usage at a young age. Both groups were treated with antihistamines and Chinese herbal medicines. Because patients were paired with their siblings, we assumed that genetic and environmental conditions were similar for both. Time to cure was the primary outcome measure, defined as the absence of atopic relapse and a score of 0 on the Scoring of Atopic Dermatitis (SCORAD) index for more than half a year.

## Results

“User” group were all elder siblings and revealed a high risk of infection. While “nonuser” group were all younger siblings. The observation period was 39.6 months (SD = 25.2 months) in “user” group, and 11.3 months (SD = 8.7 months) in “nonuser” group. A history of steroid medication increased the relative risk of incidence of staphylococcal infection (OR = 1.67, 95 percent CI: 1.06, 2.62).

## Conclusions

The steroid medication increased the incidence of the infection thereafter

